# Out of the Silence: Insights into How Genes Escape X-Chromosome Inactivation

**DOI:** 10.3390/epigenomes7040029

**Published:** 2023-11-23

**Authors:** Samantha B. Peeters, Bronwyn J. Posynick, Carolyn J. Brown

**Affiliations:** Molecular Epigenetics Group, Department of Medical Genetics, Life Sciences Institute, University of British Columbia, 2350 Health Sciences Mall, Vancouver, BC V6T 1Z3, Canada

**Keywords:** X-chromosome inactivation, escape, heterochromatin, repetitive elements, allelic expression, DNA methylation, sex differences

## Abstract

The silencing of all but one X chromosome in mammalian cells is a remarkable epigenetic process leading to near dosage equivalence in X-linked gene products between the sexes. However, equally remarkable is the ability of a subset of genes to continue to be expressed from the otherwise inactive X chromosome—in some cases constitutively, while other genes are variable between individuals, tissues or cells. In this review we discuss the advantages and disadvantages of the approaches that have been used to identify escapees. The identity of escapees provides important clues to mechanisms underlying escape from XCI, an arena of study now moving from correlation to functional studies. As most escapees show greater expression in females, the not-so-inactive X chromosome is a substantial contributor to sex differences in humans, and we highlight some examples of such impact.

## 1. Introduction

X-chromosome inactivation (XCI) occurs early in development in mammals, silencing any X chromosomes beyond one. Thus, XCI occurs in females (generally XX) and compensates for the X copy number differences with males (generally XY). XCI is a classic example of epigenetics: one X chromosome is randomly chosen to be silenced, then remains silenced through subsequent cell divisions, but is reactivated during meiosis. Perhaps unexpectedly, the inactive X (Xi) is a substantial contributor to sex differences, with more than 20% of X-linked genes examined to date continuing to be expressed from the otherwise inactive chromosome. Genes that maintain expression from the Xi are referred to as escaping XCI (or escapees), while genes that are silenced are subject to XCI. Some escapees have functional Y gametologs, but many do not. The proportion of expression from the Xi relative to the Xa differs between genes, and even between tissues for some escapees. Further, some genes are variable in their escape between cells, tissues, or individuals. In this review we first discuss the approaches that have been used to identify escapees with an emphasis on the limitations that preclude the creation of a definitive catalog of escapees. To highlight the clinical impact of these escapees, we present a few specific examples from constitutive escape genes. While improvements to the lists of genes escaping XCI are occurring continuously, we have yet to determine how a gene can avoid the chromosome-wide silencing. Variability in escape from XCI provides insights into mechanisms that allow some genes to escape from inactivation, as the same gene can be examined when it is either subject to, or escaping from, XCI. We then review studies that have examined the genomic features contributing to whether genes escape from XCI.

## 2. Identification of Genes That Escape from XCI

Generating a list of genes that escape from XCI has been challenging for many reasons, as we will discuss below. While mouse models, in particular embryonic stem cells (ESCs), offer a highly tractable system for studying XCI, turning to mouse models fails to help predict human escapees, as consistently fewer genes escape XCI in mice than in humans or other eutheria [1]. We will use the term XCI status for referring to whether a gene is subject to, or escapes from, XCI. Further, as we discuss in detail in the third section, genes may also be designated as variable escapees when escape is either tissue-specific, differs between individuals, or exhibits cellular heterogeneity (e.g., [2]). Given the clinical and biological relevance of these genes for sex differences, it is important to establish which genes escape from XCI, and in which tissues.

### 2.1. The Challenges to Determining XCI Status in Humans

**Figure 1:**
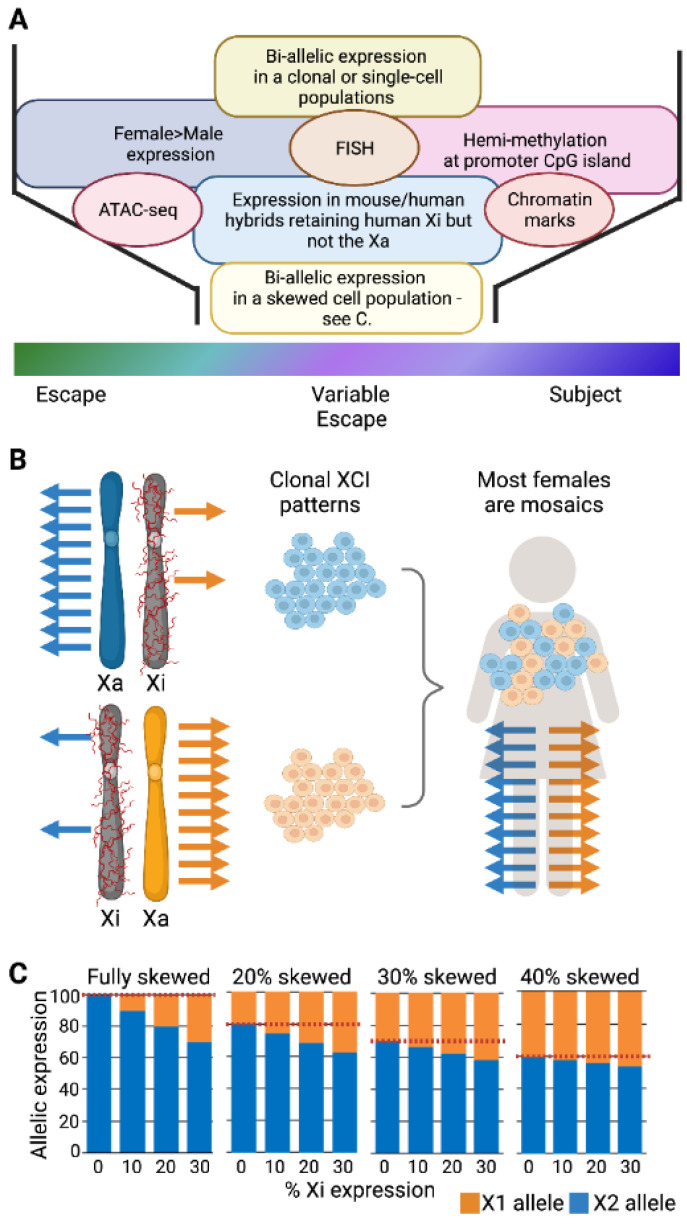
Identifying genes that escape from XCI. (**A**) Approaches to calling XCI status are discussed in text. Transcriptomic approaches include female/male comparisons, separation of the active X (Xa) and inactive X (Xi) in somatic cell hybrids or allelic analyses. FISH: Fluorescence in situ hybridization. ATAC-seq: Transposase-accessible chromatin with sequencing. (**B**) In clonal cell lines, most genes are mono-allelic in their expression from the Xa, but escapees are bi-allelic. However, most females are mosaics and express both sets of X-linked alleles (bi-allelic) independent of the XCI status of the genes. (**C**) The extent of skewing of XCI impacts the ability to estimate whether the allelic expression is coming from the Xi (red dashed line is set at zero escape which serves as the baseline from which a difference must be significant to identify escape from XCI), The studies cited in the text [21,68] used samples with greater than 20 or 25% skewing, levels generally considered moderately skewed for XCI. If there is less deviation from random XCI (such as the 30 or 40% shown here), identifying escape from XCI is unlikely to be possible.

The broadly accepted threshold for saying that a gene escapes from XCI is that the level of expression from the Xi is greater than 10% of the level of expression observed from the Xa [3]. This threshold should eliminate low-level transcriptional noise resulting in erroneous escape calls; however, biological relevance for expression from the Xi will depend on the functionality of the gene. In Figure 1A we outline the most commonly used approaches to making calls as to whether genes are subject to or escaping from XCI.

Multiple early studies relied on examining expression from the human Xi when isolated away from the human Xa in mouse/human somatic cell hybrids [3,4]. Another expression-based approach for determining whether a gene escapes from XCI is to compare its expression in females to that in males and presume female overexpression reflects Xi expression [2]. While genes that escape XCI do generally show higher female expression (reviewed in [5]), there can be many other reasons for differing female/male expression including hormonal differences, and the downstream impact of escape genes. Furthermore, as expression from the Xi is lower than the Xa, females might only show a small augmentation of expression levels relative to males, thereby restricting the approach to well-expressed genes, or those escapees with higher relative Xi expression. This line of evidence can be strengthened by extending the analyses to X-chromosome aneuploidies (e.g., [6,7]) and showing that augmented expression levels are proportional to the number of X chromosomes, not the biological sex of the individual. A recent extensive study of 176 individuals spanning 11 different sex-chromosome constitutions revealed expression changes related to the number of Xi’s for 38% of X-linked genes. Surprisingly, however, many of these changes reflected an impact of the Xi on the Xa [7], further confounding such dosage analyses. An impact on autosomal gene expression is also seen (preprint, [8]). Sex chromosome aneuploidies frequently show mosaicism in tissues, adding another challenge to such assessments in vivo and also confounding phenotype/genotype correlations (e.g., [9]).

An extension to identifying escapees by the female/male difference in expression is to study the differences in epigenetic marks associated with inactivated genes in cells or tissues with and without an Xi. The most frequently assessed and robust of these changes is the gain of DNA methylation (DNAm) at CpG-islands at the promoters of X-linked genes on the Xi [10,11,12]. Additionally, chromatin marks [10], hydroxy-methylation [13], RNA polymerase (RNAPII) binding [14], or open chromatin (Assay for Transposase-Accessible Chromatin, ATAC-seq) [15] have been used to identify the differential enrichment (or depletion) of features demarcating escape genes in females. These correlative approaches to calling inactivation status provide an indirect means to identify escape genes without directly examining Xi expression and thus require the choice of approach-appropriate thresholds. For example, DNAm studies generally require low levels of DNAm in males (used as a proxy for the Xa) and a female/male difference that is defined for the tissue by the examination of a training set of previously confirmed subject genes (see [11]). An advantage to the indirect study of DNAm is that the mark can be observed to be enriched on the Xi in tissues in which the gene is not expressed. For example, the androgen receptor (*AR*) gene shows substantial variability in expression, including minimal expression in lymphoblasts or placenta; however, differential DNA methylation at *AR* is often used to predict the skewing of XCI, including in those tissues (the HUMARA assay, e.g., [16]), although variations have been reported in some blood tissues [17]. An additional appeal to DNA-based assays is that DNA is often more stable for recovery from tissues; however, a direct assessment of expression from Xa and Xi avoids confounding co-correlations. The direct visualization of expression is possible on a gene by gene basis using RNA fluorescent in situ hybridization (FISH) if the point of transcription is strong enough to be visualized as mono-allelic or bi-allelic (e.g., [18]). For a broader survey, the transcriptomic analysis of expressed polymorphisms allows for the assessment of many genes simultaneously, as discussed below.

Allelic expression studies of X-linked genes are limited by the availability of expressed polymorphisms, and further challenged by the random nature of XCI leading to each X being active in a subset of cells. In mice, such allelic studies can be optimized by using crosses between evolutionarily distant strains (F1 crosses) to increase the number of allelic differences between the two X chromosomes. The use of mutations or transgenes to enforce inactivation of only one of the two X chromosomes (e.g., [19,20]) further improves the informativity. In the large GTEX survey of human tissues, a female was found with completely skewed XCI, allowing for the direct allelic discrimination of expression from the Xi. Only 186 informative genes (of the ~1000 X-linked genes) were identified with sufficient expression to allow for the robust determination of inactivation status, revealing 23% of informative genes to have some bi-allelic expression [2]. In contrast, in the Nesterova et al. study in mouse ESCs, 500 genes were informative with at least 10 reads [20]. While partially attributable to the broader expression in ESCs, this comparison highlights that the limited number of expressed polymorphisms in humans reduces the number of genes that can be examined per individual. To acquire broad coverage of X-linked genes, it is thus necessary to analyze the transcriptomes of many individuals.

While there are transcriptomic studies of large groups of humans, most females are mosaics (see Figure 1B) and express both sets of X-linked alleles independent of the XCI status of the genes. Only rarely will individuals have completely skewed XCI. Thus, studies have undertaken bioinformatic estimations of the deviation in allelic expression for an individual gene from the average allelic ratio calculated for the chromosome to estimate if there is expression coming from the Xi. These deviations will be small if the extent of skewing is not extreme, as illustrated in Figure 1C. As an example, to assess bulk RNA sequencing (RNA-seq) from the Geuvadis cohort, Sauteraud et al. developed an R package called X-Chromosome Inactivation for RNA-seq (XCIR) and used a training set of 177 genes commonly subject to XCI to determine the baseline average level of allele-specific expression, thus reflecting the level of X-inactivation skewing in each sample [21]. With RNA-seq from 217 female lymphoblast cell lines (LCLs), they identified 136 samples with a skewing of XCI greater than 25:75. The use of phased genomes from UK Biobank DNA-seq information allowed for the integration of multiple SNPs per gene and improved allelic read depth by almost 50%. There were 215 genes with calls in at least 10 individuals, and escape was called when Xi expression was observed in greater than 75% of individuals (20 genes or 9.3% of genes categorized); similarly, genes were called subject to inactivation if less than 25% of females had Xi expression (165 genes or 77% of analyzed genes), and the variable category was for genes between those thresholds (30 gene or 14%). A further advantage of using large human datasets is that they often have extensive phenotypic information, and the X chromosome is notoriously neglected in genome-wide association studies [22]. The knowledge of XCI status can facilitate association studies for the X. Indeed, Sauteraud et al. found heritability enriched in escape genes with Y gametologs, which they suggest could reflect the stricter evolutionary constraint of those genes [21].

The percentage skewing of which X chromosome is inactivated is normally distributed, with approximately one quarter of random peripheral blood samples showing incidences of greater than 70%, but only 8% showing a skewing of XCI greater than 80%. The proportion of extreme skewing increases with age and for carriers of X-linked disease in blood [23,24,25]. The likelihood of extreme skewing will also be influenced by the number of epiblast progenitors contributing to the tissue [26] as well as the amount of migration within the tissue. For example, placenta shows large patches of cells with the same X inactivated, such that small tissue samples can capture a clonal population for RNA-seq [27]. For other tissues, even small samples are generally mosaic and capturing the expression from each Xi may require analysis of single cells.

Single-cell RNA sequencing (scRNA-seq) is becoming more widespread in studies of human tissues, and these data can be gleaned for information about XCI status. In order to distinguish bi-allelic expression from mono-allelic expression, having the phased X chromosome genotype is helpful as widespread mono-allelic expression is routinely observed in single cell transcriptomes [28]. Additionally, transcriptional bursts make allelic ratios unreliable unless the gene is sufficiently well expressed and the results are aggregated from multiple cells. Furthermore, any cell doublets need to be excluded [29]. In mice, deep single-cell RNA-seq allowed for the assessment of 576 X-chromosomal genes from a B6-Castaneus cross throughout development [30]. In humans, the scRNA-seq of cell lines from four individuals in the GTEX study yielded 41–98 genes with enough informative reads to call an XCI status per individual; in aggregate, 165 genes could be assessed. Additionally, the use of scRNA-seq allowed for the detection of cellular heterogeneity and the influence of genotype on XCI status between the two X chromosomes within the same individual [2]. Although further optimization and unified thresholds are still needed, these methods have enabled a greater ability to call which genes escape XCI, particularly across cell types and differentiation.

### 2.2. Which Genes Escape XCI?

XCI is a chromosome-wide silencing event that radiates from the X-inactivation center, where the XCI-initiating long non-coding RNA, XIST, is expressed. XIST acts as a modular integrator that recruits multiple additive silencing pathways, including SPEN, PRC1, PRC2, and SMCHD1 [31]. Through protein–protein aggregation the XIST-binding proteins and their partners condense and silence the Xi [32,33]. The degradation of the Y-linked gametologs that created the need for dosage compensation occurred episodically, with evolutionary strata discernable from when X/Y recombination ceased [34]. If Y gametologs persist, then the silencing of the gene on the Xi would lead to XY males expressing more gene product than XX females. Thus, biologically it makes sense for X-linked genes with functional Y gametologs to escape from XCI. The pseudoautosomal regions (PAR1 and PAR2) are equivalent between the X and the Y as they continue to undergo homologous recombination during male meiosis (albeit at different rates). All of the Xp PAR1 genes examined escape XCI, while for the Xq PAR2 region, the distal genes escape XCI, yet more proximal genes are subject to XCI (and also silenced on the Y chromosome) [35,36,37,38]. Beyond the PARs, genes with Y homology continue to be more likely to escape from XCI. While the Y chromosome retains only 3% of the ancestral X chromosomal genes, there are some genes that retain X and Y versions across many species [39]. Of these 36 ancestrally conserved genes, 17 remain X/Y pairs in humans (excluding SRY/SOX3), and of these, 12 escape XCI. Of the 18 that have lost the Y gametolog in humans, 8 escape XCI [40]. It has been suggested that the ancestrally-conserved genes were exquisitely dosage-sensitive either directly because of function (they were enriched for function in transcriptional control) or through participating in large dosage-sensitive complexes [41]. There is a 3.5 Mb region on the X proximal long arm that was transposed from the X to the Y approximately 5–6 million years ago and contains the escapee *PCDH11X*, again emphasizing that evolutionarily recent additions to the X and Y chromosome are not yet silenced by XCI [42], perhaps pointing to a need to acquire sequence changes to assist the spread of XCI into new regions. The genes adjacent to the PAR1 in the more recent evolutionary strata also escape XCI. Indeed, there is a strong enrichment for genes that escape XCI to be on the human Xp region that was added to the X chromosome after divergence between eutheria and marsupials [3]. Variable escape genes tend to enrich between escape and subject domains [40]. Interestingly, the escapees from Xp tend to be found in clusters of multiple escape genes, while the fewer escapees on Xq tend to be solo (reviewed in [43]). This raises the question of whether some genes escape from XCI because they are ‘bystanders’ to genes that have signals to escape from XCI. Such bystanders may lack a strong ‘escape’ or ‘subject’ signal and thus derive their inactivation status from the domain in which they are found. For a more complete discussion of which genes escape from XCI and the evolutionary drivers that may provide the basis for escape from XCI, see our recent review [44].

## 3. Clinical Implications of Escapees

As mentioned above, many escapees are considered to be dosage-sensitive. The myriad of clinical presentations of Turner syndrome (TS) in 45,X individuals have long been attributed to the missing escapees from a second sex chromosome (see [45] for a recent review). Similarly, the features of Klinefelter syndrome (KS; 47,XXY, or more rarely, 48,XXXY or 49,XXXXY) and also 47,XXX (or 48,XXXX or 49,XXXXX) individuals are believed to be associated with increased dosage of escapees [5]. Until recently, the only gene definitively linked with these syndromes was the PAR1 gene *SHOX*, accounting for both the short stature in TS and tall stature in KS and triple X individuals [46,47]. The RNA-seq of fibroblasts and LCLs in 176 aneuploid individuals revealed 10 genes that may drive phenotypic impacts of variation in the Xi copy number, including the PAR1 genes *SHOX* and *SLC25A6* [7]. Many of the identified genes have also been linked to disease phenotypes in 46,XY and 46,XX individuals. Unless otherwise stated, the escape genes below were among those identified by San Roman et al. [7].

The X chromosome is evolutionarily unique because it spends more time in females yet is more vulnerable to selective pressure in the hemizygous male (see [44]). X-linked disease is therefore more common in males, and for severe mutations in critical genes, there may be no affected males due to early lethality. Heterozygous (carrier) females will be mosaics due to having the mutation on the Xa or Xi in different cells. For genes subject to XCI, this mosaicism can manifest in patchy expression, leading to no phenotype or a reduced phenotype compared to affected males (see [48]). The skewing of XCI in females can either reduce clinical effects (if the pathogenic allele is selected against) or increase the pathogenicity of the allele (by chance, or if the other X chromosome has stronger selection against it). In fact, frequently, the extreme skewing of XCI often reflects selection against a detrimental allele being on the Xa. For genes that escape XCI, the mosaicism will be less distinctive, as both Xs will express the gene; however, the Xi generally shows less expression than the Xa, and so, cells with the mutation on the Xa may be more detrimentally affected.

To date, 199 X-linked intellectual disability syndromes (ID) linked to 162 genes have been identified [49]], including the escape gene *DDX3X*. *DDX3X* is responsible for ID in both males and females: the loss of function mutations are male-lethal, while hypomorphic mutations lead to affected males and carrier females [48]. Mutations in the escape gene *USP9X* follow a similar pattern [48]. Interestingly, mutations in both genes have been associated with tumor progression and worse cancer prognosis, particularly in males [50,51]. This lends credence to the EXITS hypothesis, which states that females are protected from some cancers through the escape of X-linked tumor suppressor genes, and is one explanation for the male predominance of many cancers [52].

An interesting pair of escapees are the epigenetic modifiers *KDM5C* and *KDM6A* which have also been linked to both male-biased cancers [52,53,54] and ID [55,56]. In addition, both genes have been associated with complex disease states. The use of XX males and XY females in the Four Core Genotypes sex-reversed mouse model have delineated effects of chromosomal sex versus sex hormones in mice [57], and have revealed that *Kdm5c* dosage directly impacts adiposity. BMI in humans is also affected by *KDM5C* dosage [58]. *KDM6A* has been implicated in the observed female-bias in Alzheimer’s disease [59], although not all studies have identified *KDM6A* expression as a contributor [60]. Interestingly, *KDM6A* and *KDM5C* have recently been suggested to play opposing roles in autoimmune responses in females [61], consistent with the finding that increased *Kdm6a* expression leads to a greater activation of neuroinflammation signaling pathways in mice [62].

Sex differences in immunity are well established, although not fully understood (see [63] for a recent review). Females generally have a better immune response against infection; males have had significantly worse disease outcomes for COVID-19 [64], possibly through greater T-cell response in females [65]. Variable escape of *TLR7* (a gene not expressed in the tissues studied by San Roman et al.) has been observed in both T-cells and B-cells, and is thought to contribute to the dramatic sex-bias of autoimmune diseases in women [66,67].

## 4. Variability in Escape from XCI

As different studies use different methods to call XCI status, often with different thresholds, and in different sample sets, it is not surprising that they generate different lists of genes that escape from XCI. Multiple studies have tried to aggregate these datasets into standardized calls (e.g., [2,40,68]). Concordance is high for a subset of genes, sometimes described as constitutive escape genes, while variability seems to be an inherent feature of other genes, sometimes described as facultative escape genes. As studies examine larger numbers of individuals, it has become clear that occasional expression from the Xi can occur, and even genes with robust classical evidence for XCI are called as escape in some individuals or tissues in some studies. For example, the Duchenne muscular dystrophy gene, *DMD*, was reported as variably escaping XCI in two recent studies [21,68] despite protein expression in heterozygous females showing cellular mosaicism, and carrier females generally showing milder symptoms unless XCI is skewed towards silencing the intact allele [69]. Escape from XCI was detected for *DMD* in tissues with much lower expression levels than seen in muscle. Low levels of expression might reflect the lack of stringent XCI regulation; but may also reduce the accuracy of distinguishing escapees.

**Figure 2:**
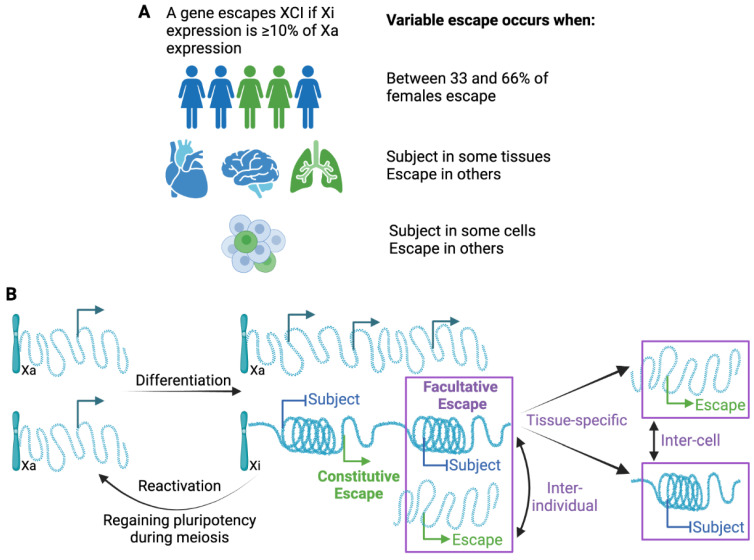
Variable escape from XCI. (**A**) Escape can vary at individual, tissue, and cellular level; however, thresholds for such variability differ between studies. (**B**) Variability suggests instability in either silencing or escape when observed between cells within the same tissue of the same individual (inter-cell). Inter-individual escape could reflect genetic differences, but to date only limited evidence for genetic contributions has been found. Tissue-specific escape suggests that genes reactivate after XCI as the escape is generally only in a single or few tissues; however, silencing could also occur in a tissue.

As the number of studies and approaches have increased, so has the identification of variability, not only between individuals but between tissues and even cells within a population [2]. Adding to the variability between studies, standardized thresholds have not been adopted regarding how frequently a gene must differ from the more common XCI status call in order to call it variable. The first extensive study identifying variable escape genes used both somatic cell hybrids and allelic expression, and called variable escape when three to six of the nine Xi-containing hybrids showed significant expression, and thus, a threshold of 33–66% of samples is often used ([3], see Figure 2A). Different thresholds might be appropriate for different studies, but it is important to know the underlying threshold when describing variability. In addition to being aware of the variability between studies, intrinsically variable genes may provide insights into the mechanisms underlying escape from XCI. As shown in Figure 2, while inter-individual differences likely are present from the time of XCI, tissue-specific escape from XCI may often reflect the reactivation of previously silenced genes. While XCI is an impressively stable epigenetic silencing event, reactivation, particularly in the concept of cancer or aging, could contribute to sex differences.

### 4.1. Inter-Individual Differences in Escape from XCI

Variability between individuals for escape from XCI has been known for decades [70,71]. Individuals may differ in escape status due to genetic differences or epigenetic differences acquired during their lifespan. One study approached this question using bulk RNA-seq data from the TwinsUK dataset [68]. In total, 248 individuals with skewed XCI (based on XIST allelic ratio > 0.8 or <0.2) were included, representing 14–30% of the lymphoblast cells, adipose tissue, and skin cells tested. They identified both constitutive and tissue-specific escapees, using twins to identify both genetic and environmental influences on escape [68].

There are some interesting candidates for genetic contributors to escape, although none have been shown to have a large contribution. Using a combination of epigenetic marks to predict variable escapees, Balaton performed an association study and identified multiple contributory loci; however, no locus was sufficient to predict XCI status, and indeed, overall, their relative contribution was small [10]. Three autosomal loci (mapping to *SMCHD1*/METTL4, *TRIM6*, and *ZSCAN9*) were shown to impact the DNAm level of some X-linked variable escape genes [72]. SMCHD1 has been shown to be critical for DNAm and the silencing of a subset of mouse X-linked genes [73]. As mentioned above, in the GTEX data, differences were seen between the two Xs for a gene’s likelihood to escape, implicating *cis*-acting factor(s) [2]. scRNA-seq from five females identified 22 robust escapees, which exhibited a broad range of allelic ratios, with the distribution differing between genes. When the ratios were averaged across the genes per cell the resulting inactivation score was seen to correlate independently with cell cycle and to a lesser extent with *XIST* expression [29]. However, no individuals have been observed with either an unexpectedly high or low proportion of variable escape genes being active, suggesting that the per-cell effect does not translate to a per-individual effect [3,74].

### 4.2. Tissue-Specific Variable Escape from XCI

The ability of a gene to be subject to XCI in some tissues and escape in others suggests that for these genes the unique epigenetic environment of the tissue type is the most important feature allowing for the escape from inactivation. Many genes are tissue-specific in that they are expressed in only one (or a limited number) of tissues. If these genes are also escapees, then they are sometimes referred to as tissue-specific escape genes; however, we consider them instead to be tissue-specific genes escaping XCI, a subtle but important difference. To distinguish variable escape as being tissue-specific, numerous tissues of the same individual need to have been examined. GTEX includes a variety of tissues from both males and females, but only one completely skewed female was extensively studied [2]. Nonetheless, they did observe that the Kallman syndrome gene, *KAL1*, demonstrated bi-allelic expression indicative of escape from XCI solely in the lung, and that such expression results in a sex difference in *KAL1* expression (in the lung). Overall, they found few genes that escaped XCI in a tissue-specific manner, and a new preprint suggests that with the addition of two more fully skewed females, there is still only limited tissue-specific escape (preprint, [75]). In contrast, in the Twins-UK study, 23% of genes were called as having tissue-specific escape [68], with 49 genes called as single-tissue escapees, although only three tissues were examined. As this study used incompletely skewed XCI, the thresholds chosen may have contributed to the higher proportion of tissue-specific calls. Some of the variability in inactivation patterns may lie in differences in the X-inactivation machinery itself, as B cells (discussed in more detail below) have been shown to have different XIST-interacting partners [67]. As with all tissue-specific expression, the transcription factor (TF) componentry of the cell likely also plays an important role.

Genes that are variable in their escape from XCI are more challenging to robustly define, at least partially because thresholds have not been clearly delineated, as outlined in Figure 2A. Variable escape could reflect less stable silencing through gene reactivation. This is likely to be the case for tissue-specific genes in which expression is seen in only a restricted set of tissues (see Figure 2B). Alternatively, when escape is variable between individuals, it may reflect differences in the ability to establish XCI early in development. For variability between cells within the same tissue, we could be seeing a loss of silencing in some cells, or gain of silencing in some cells (see Figure 2B). For TIMP1, we previously demonstrated that promoter acetylation predisposes towards an unstable methylated and expressing state that can resolve to either a stable, silenced, and methylated, or expressed and unmethylated state [71,76,77].

### 4.3. Reactivation of Inactivated Genes

The reactivation of the entire X chromosome occurs during reprogramming—either naturally during gametogenesis or during a culture-enforced return to naivety in pluripotent stem cells (PSCs). Mouse ESCs have two Xa’s, one of which inactivates upon differentiation, making them an ideal model system for studying the initiation of XCI. Human ESCs, on the other hand, have an Xi and Xa when initially derived, modelling a slightly later stage of development that is epiblast-like. Further complicating the use of human PSCs in therapeutic approaches or to study human XCI, there is erosion of the Xi; with loss of XIST, chromatin modifications and a progressive return to bi-allelic expression observed (reviewed in [78,79]). The reactivation of the Xi in PSCs is far more extensive than the limited reactivation seen in somatic cells, but interestingly the reactivation is reported to spread from existing escape genes [80]. In somatic cells it has long been believed that inactivation is extremely stable [81], and that reactivation of individual genes occurs only at very low to unmeasurable levels unless Xi-features are removed (discussed further below, and see [82]). Using assessments of skewing across multiple tissues, the timing of XCI in humans has been demonstrated to occur before lineage commitment [25]. Therefore, tissue-specific escape suggests that genes reactivate (see Figure 2B), and indeed, reactivation has been proposed to lend resilience to female brains during differentiation (preprint, [83]).

Surprisingly, it appears that one mechanism allowing for the reactivation of genes is loss of the XIST RNA from the Xi. Auto-immune diseases are strikingly sex-biased, with over 80% of systemic lupus erythematosus (SLE) patients being female. There are numerous X-linked genes known to be important in immune disease, and there is now strong evidence for the dynamic regulation of the Xi in B cells, which is disrupted in SLE [84]. XIST is constitutively expressed, and localizes to the Xi in most somatic cells; however, in quiescent B (and other immune) cells, despite ongoing XIST expression, localization is only observed upon immune activation. Furthermore, there is a B-cell-specific XIST interacting protein, TRIM28, involved in RNAPII pausing at X-linked promoters. The loss of XIST localization leads to loss of Xi modifications and the reactivation of some genes, including *TLR7*, an important player in SLE [85,86,87]. In B-cells, 36 XIST-dependent genes were identified and seen enriched for both immune-related gene functions and also variable escape from XCI [67]. XIST depletion or knock-out studies have revealed XIST-dependent genes in multiple tissue types, including cancer cells [67,88,89,90]. These findings of reactivation, both XIST-dependent and underlying tissue-specific escape, further highlight that the inactivation status of genes is a continuum rather than a clear categorization.

### 4.4. Impact of Age

Tissue-specific escape and XIST-dependent gene reactivation emphasize that XCI is not as stably locked into silencing as was once thought, raising the question as to whether the fidelity of XCI could be reduced with age. In an early study examining the subject gene *HPRT1* (which can serve as a selectable marker for reactivation in heterozygous cells), no substantial increase in reactivation was seen with aging [82]. In mouse hematological cells, the loss of *Xist* causes hematological malignancies, and aging disrupts laminar association, correlating with the variability of expression, DNA hypomethylation, and increased chromatin accessibility on the Xi [91]. Females show greater risk for DNA damage and cellular senescence [91], and it is possible that the stability of XCI and escape gene expression might be involved. To date, human studies of XCI and aging have focused on DNA methylation, which is a key lock to the stable silencing of X-linked genes [92] and is known to change with age on the autosomes (see [93,94]). A number of recent studies have found that several hundred CpG sites have significantly changed DNAm (both gains and losses) correlating with age in a sex-dependent manner [95,96,97]. There is limited overlap between the studies, which covered different age ranges, consistent with a new study, which suggests that the Xi accumulates epigenetic variability with age [98]. Interestingly, Li et al. identified the loss of DNAm to be enriched inearescapees [96]. The Xi is often described as hypermethylated; however, the increase in DNAm is found at promoters—particularly CpG-island promoters—while the gene body and intergenic regions are actually slightly less methylated on the Xi than the Xa, perhaps consistent with a general lack of transcription [11]. Overall, the stable silencing of X-linked genes is believed to be due to overlapping silencing pathways, but how much these can be disrupted during aging remains a topic of exploration.

## 5. Elements Associated with Escape from XCI

The unique physical structure of the Xi was first observed as a nucleolar satellite [99]; the determination that this was likely an X chromosome [100] contributed to Mary Lyon’s 1961 hypothesis that one X chromosome was inactivated in mammalian females [101]. Since that time, multiple epigenetic features have been found to differentiate the Xi from the Xa. In general, the silenced Xi is characterized by marks associated with heterochromatin, such as H3K9me3, H4K20me1, H3K27me3, and the macrohistone H2A (reviewed in [43]). Escape genes on the Xi are associated with active marks, such as H3K4me3, and acetylation, although both escape promoters and enhancers still have more H3K27me3 and less H3K27ac than their Xa counterparts [10]. This is not surprising considering that escape genes generally have lower expression from their Xi allele, and further illustrates that the two chromosomes are not equivalent, even at escape genes. While epigenetic marks have been useful to distinguish escape and subject genes in somatic cells, the heterochromatic marks associated with XCI are avoided by escapees, underscoring that genomic features are likely critical components of how genes establish escape from XCI.

### 5.1. The Physical Structure of the Inactive X Chromosome

Differences in the chromosome territory have long been noticed between the smooth spherical Xi and the flatter, larger, and more irregular Xa territory [102,103]. However, the overall chromosome compaction is only 1.2× greater for the Xi than the Xa, with wide variation at different chromosomal locations [104]. Chromatin domains of 1 Mb show similar compaction between the Xa and Xi, with differences only appearing at the 20 Mb level. However, that level of compaction does not appear to be correlated with gene density, transcriptional activity, LINE (long interspersed nuclear element) content, or histone modifications on the Xi [104]. This has been a surprising finding, as Xi compaction has classically been thought to be an important driver of Xi regulation (see [105] for a detailed review).

Recently, the advent of chromatin conformation capture techniques, together with reduced sequencing costs, have enabled investigations of physically-interacting topologically-associating domains (TADs) [106,107]. The Xi is generally lacking in TADs except around escape genes; instead, the macrosatellite repeat *DXZ4/Dxz4* acts as a hinge for the Xi, forming a large bipartite structure [108,109,110]. The role of *DXZ4/Dxz4* in XCI has recently been reviewed by Loda et al. [111]. While the repeats impact megadomain formation and TADs on both the mouse [112] and human [110] Xi, they apparently have only a minor role in regulating XCI [111]. Deleting *Dxz4* from the mouse X prior to XCI resulted in a small decrease in variable escape upon differentiation [112], but when the deletion was made in somatic cells, such an effect was not observed [113]. Similarly, the somatic deletion of human *DXZ4* did not have an effect on silencing or escape during XCI maintenance despite a drastic change in chromosome superstructure [110]. The limited effect of *DXZ4/Dxz4* deletion on escape is notable, as many escapees in both mouse and human had been previously observed to roughly correspond with the limited TADs on the Xi [112,114]. The differences between studies could reflect XCI initiation versus maintenance; however, a more recent study showed that genes are silenced prior to or accompanying TAD loss on the mouse Xi, rather than as a result [115]. Although some escape domains are conserved between species [1], variable escapees are often regulated at the gene-level in humans [10].

A study of 15 X-linked genes by RNA-FISH postulated that the human Xi may be organized into a gene-rich outer rim and a gene-poor core [18]. This finding supported an earlier FISH study showing that the mouse Xi contained an internal Xist RNA domain devoid of RNAPII and TFs, with escape genes found on the Xi periphery [116]. However, electron microscopy and higher resolution FISH studies have since shown that the escape genes are found throughout the Xi territory, and that the physical Xi is “sponge-like” to allow for some accessibility by RNAPII and TFs [104,117,118]. Indeed, the loss of RNAPII occupancy at the Xi does not appear to be driven by RNAPII accessibility *per se* [119].

Recently, Cerase et al. have proposed that phase separation is a driver of XCI [120,121]. It is well established that there are fewer Xist molecules bound to the Xi at any one time than there are silenced genes [32,118,122,123]. To facilitate whole-chromosome silencing, it seems likely that Xist interacts with its binding partners in a non-stoichiometric manner. The Xist domain has properties resembling liquid-liquid phase separation [120], and multiple groups have shown that large assemblies of proteins coalesce around each Xist focus [32,33,124]. These protein assemblies include SPEN (SHARP), CIZ1, and components of PRC1/PRC2, and might enable Xist-independent maintenance. Phase separation may also help answer long-standing questions of how chromosomally-proximal genes can have different XCI statuses. However, it should be noted that not all recent findings are necessarily consistent with phase separation on the Xi (see [119]). In addition, phase separation experiments to date have focused on mouse cells. While it seems likely that a similar mechanism would exist for the human Xi, there are differences in XIST/Xist domains between the two species [125] that may be reflected in phase separation. Lastly, there has been little exploration of the implications of phase separation for escape genes.

### 5.2. Identifying Genomic Features Associated with Escape from XCI

There is evidence supporting both regional features as well as gene-specific regulatory elements enabling escape. As mentioned above, mouse is an outlier for the number of escape genes. Only 3–7% of mouse genes are expressed from the Xi, in contrast to humans and most other species examined, which have 10–20% of genes (excluding PARs) escape from XCI [1]. The arrangement of most human escape genes into larger blocks of genes clustered on the short arm of the X has been suggestive of domain regulation, while most of the mouse escapees are singletons, suggestive of local regulatory elements driving expression. A subset of escape genes conserved across all species is supportive of more proximal or gene-specific regulation as a factor; however, the escape genes that are discordant in XCI status between species often switch status as a block, indicating some domain regulation is also involved in their expression [1]. In mouse, a greater proportion of the open chromatin on the Xi is found close to promoters relative to what is found for the Xa, suggesting that escape may be regulated through promoter-proximal sites [112].

The strongest evidence for intrinsic escape elements in the DNA sequence in or near escape genes is derived from transgene studies. A series of four random X-linked integrations of bacterial artificial chromosomes (BAC) containing the mouse escape gene *Kdm5c* revealed that the gene was able to escape from XCI outside of its endogenous location on the Xi [126]. *Kdm5c* escape was reproduced in our independent transgenic mouse study that integrated the same 174 kb BAC at a targeted integration site 5′ of the X-linked *Hprt* locus, which is normally subject to XCI [127]. The vast majority of transgenes docked at this locus become subject to XCI when integrated onto the X chromosome, further implicating unique features at *Kdm5c*, allowing for ongoing expression from the Xi [128,129]. As mouse is a common model organism for XCI and escape but has considerable differences in escape gene number and distribution compared to human, we generated transgenic mice with a 158.5 kb human BAC containing the primate-specific escape gene *RPS4X*, variable gene *CITED1*, and subject gene *ERCC6L* integrated at *Hprt*. The XCI status of all three genes were recapitulated across several tissues and developmental time points, establishing that intrinsic elements within the *RPS4X* BAC share recognizable properties between mouse and human, and that some conservation of escape mechanism is likely [127,130].

**Figure 3:**
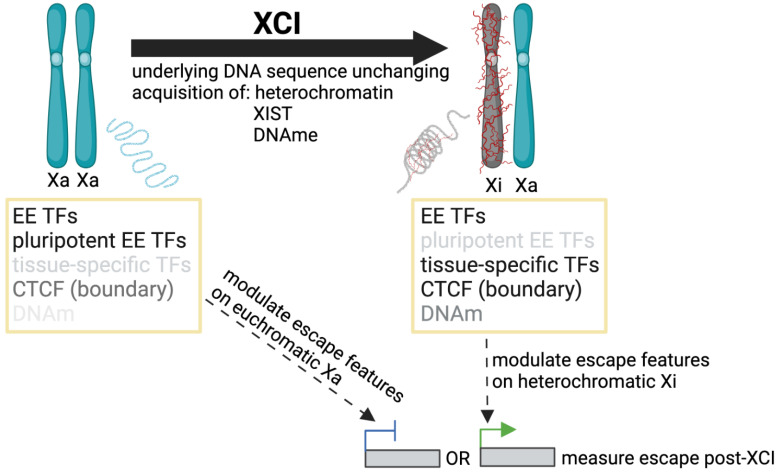
Studying mechanisms of escape pre and post XCI. The developmental time point at which we search for non-motif based escape elements could determine the significance or availability of these features for enrichment studies (i.e., pluripotent transcription factors (TFs) that are not as abundant in somatic cells); similarly, the time at which we modulate escape, pre or post XCI (integrate escape transgene, knock down TFs, manipulate DNA methylation (DNAm), or alter chromatin boundaries, etc.) could have different outcomes on escape measured post XCI. Additionally, constitutive and facultative (or reactivated) genes may show different sensitivities to the same factor modulation.

Multiple studies have identified features differentially enriched at escape and subject genes, with key contributions highlighted in Table A1. No single feature or combination of factors has yet been characterized to accurately predict the XCI status of all genes, but potential contributors, as well as the methods used to detect and refine them, are summarized below. A limitation to such studies is that the challenges in assessing which genes escape from XCI (Section 2) have ramifications in the search for escape elements themselves; the criteria for determining XCI status impacts the list of genes used to call enrichment. XCI status can be newly generated for the model system being studied, and/or referenced from a consensus list of previous studies. Similarly, data used for enrichment around escape genes can be generated in the same model system as the list of escape genes or analyzed from previous studies where tissue type or developmental time point may be different. The underlying genome is usually unchanging for sequence motifs; however, one should consider that different TFs may bind at escapees prior to XCI as opposed to after (as shown in Figure 3).

Repetitive elements were some of the earliest genetic sequences to be linked with XCI, with the hypothesis that LINE elements could act as waystations for the spread of inactivation given their two-fold enrichment on the X [131]. LINEs have since been found to be enriched in regions of genes subject to inactivation, and depleted around genes escaping from inactivation (see Table A1). Notably, this is also true for autosomal genes as demonstrated by studies in human looking at spread of XCI in X;autosomal translocations [132,133], and in mouse with inducible *Xist* transgenes on autosomes [134], which all identified a strong correlation between silencing and the presence of LINE elements in autosomal DNA. In contrast to LINEs, SINEs (short interspersed nuclear elements; Alu in human and B elements in mouse) are less prevalent on the X than autosomes but are enriched around genes escaping from XCI in comparison to genes that are subject to it (see Table A1). As the density of Alu elements around escape genes is at parity with the genome average in the X;autosomal translocations, regions of subject genes are relatively depleted for SINEs [132].

Alu elements can modulate gene transcription by contributing TF binding sites [135], including CTCF [136,137], which may explain why Alu elements are found in close vicinity to escape genes. A 2005 study examining the transition region between several escape genes and their subject neighbours was the first to suggest that CTCF might play a role on the Xi in maintaining both inactivated and escape domains [138]. Following this, numerous studies have expanded the search for CTCF binding across the X and found an enrichment of peaks at both the promoters of escape genes and the edges of escape domains, suggesting a role in both transcriptional and domain regulation (detailed in Table A1). CTCF enrichment was absent for autosomal genes escaping XCI (initiated by inducible *Xist* transgenes), suggesting an X-chromosome specific role for CTCF in mediating escape [134]. After reporting that convergent arrays of CTCF binding sites insulate escape genes in mouse cells, a recent study discovered that deletion, but not inversion, of the 5′ CTCF boundary region of variable escape gene *Car5b* resulted in the loss of escape (preprint, [139]). Conversely, a 3′ boundary truncation of the previously mentioned *Kdm5c* BAC resulted in spread of escape to neighbouring genes downstream of its integration site, giving an opposite effect to the *Car5b* locus [140]. Flanking cHS4 insulators with demonstrated CTCF sites and boundary activity [141] are not sufficient on their own to protect *GFP* transgenes from silencing on the Xi [129,142]. However, a recent study reported that artificial CTCF tethering to a methylation-edited *MECP2* locus enhanced *MECP2* reactivation more than just promoter demethylation alone [143]. Therefore, CTCF appears to play a role in both protecting and enhancing escape, but additional features are necessary to initiate it.

A handful of studies have started to analyze the enrichment of other TFs at escape genes with interesting preliminary results (see Table A1), including the observation that while certain factors are enriched at escape compared to subject genes, escape genes are on par with the genome average in overall number of TFs binding at the TSS [142]. As the *RPS4X* gene was previously shown to escape from XCI on a BAC, and its TSS region was enriched for top escape TFs, our group generated several combinations of *RPS4X* promoter and genic elements to characterize the minimal region necessary for escape from XCI. The *RPS4X* promoter (driving a reporter gene) flanked by cHS4 insulators was surprisingly unable to escape from XCI; however, swapping the reporter gene for additional *RPS4X* sequence was able to restore escape, suggesting that the transcription of escape elements beyond an insulated escape gene promoter is important.

Several mouse studies looking at gene expression prior to XCI have noted that escapees tend to have higher initial expression than subject genes, and that a lower expression level leads to more efficient silencing on the X [134,144]. Epigenetic marks post-XCI can be used to identify escape genes, but it is not known whether they are causative of escape or are caused by the ongoing transcription of the gene. However, autosomal studies with translocated or inducible *Xist* analyzed pre-existing marks of heterochromatin on autosomal genes and found that they correlate with whether or not genes will be subject to XCI [132,134]. The understanding of the modifications to RNA and their impact have lagged behind the surge in the characterization of the DNA and histone modifications comprising the epigenome; however, a recent study reported reduced levels of the destabilizing mark *N*^6^-methyladenosine (m^6^A) in transcripts from the X chromosome in comparison to autosomes [145]. While XCI compensates for the difference in X dosage between XY males and XX females, Ohno also proposed that dosage compensation from the ancestral X to a Y bearing only a fraction of the ancestral genes would require an approximately two-fold upregulation of the X chromosome ([146], reviewed in [44]). While reduced m^6^A is a candidate for explaining X-chromosome upregulation, genes that escape X-inactivation did not show a higher number of m^6^A motifs than silenced X-linked genes, suggesting that such marks do not distinguish escapees.

X-wide enrichment studies propose significant candidates for elements regulating escape from XCI and help to explain how the active genes on an inactive X might be evolving unique properties in comparison to subject and autosomal genes. It will be interesting to see how this list of features develops as new X-wide data become available, and enrichment analyses are expanded across a wider range of cell lines and developmental time points. Functional studies on a limited number of genes offer a way to directly test features, discover new ones unique to a subset of escape, and explore how these candidates might be working together. Like the enrichment studies, they could also be tested pre and post XCI to weigh the importance of factors during initiation and maintenance. Knockout/down studies could be utilized to reveal more factors involved in escape but face challenges if they interfere with cell survival in a key developmental window. A mouse study on imprinted (paternal X-inactivation in rodent extra-embryonic cells) escape suggested that genes escaping from XCI do so by using the same regulatory sequences as their active X copy [147], which further complicates assigning an escape-specific role to an element that is also necessary for the basic transcription of the gene.

## 6. Conclusions

In conclusion, variability—both inter-individual and tissue-specific—point to the involvement of TFs and chromatin regulators acting at the gene-level. On the other hand, domains of escapees argue for regional contributions. This contribution may derive from the depletion of LINEs reducing the capacity to spread silencing. In addition, the enrichment of Alus may bring in essential components for transcriptional activation, including CTCF, which is also the prime candidate for establishing boundaries between escape and subject domains. The involvement of the X inactivation machinery, including a potential role for phase separation, in the ability of genes to escape from XCI remains to be determined. Transgene studies, now with a smaller plasmid-based recapitulation of escape, demonstrate that local elements are sufficient for escape and impressively that human escape signals are recognized by mouse. Such approaches will allow for the dissection of which specific combination of elements is sufficient to mark a gene to avoid the silencing of X-chromosome inactivation.

Understanding how genes escape from XCI has both foundational and therapeutic implications. As exceptions to the normal spread of silencing, escapees reveal the importance of new players in the process of XCI—as evidenced by the finding of TRIM28 in lymphoid XCI (as discussed above, [67]). Indeed, with over 20% of human genes showing some expression from the Xi, we will not fully understand XCI until we can explain escapees. Being able to protect an escapee from silencing could be applicable to being able to protect transgenes from silencing, both for experimental modelling and gene therapy. Additionally, the reactivation of genes from the otherwise inactive X is being explored as therapy for Rett syndrome, in which affected females are heterozygous for mutations in the *MECP2* gene subject to XCI (as discussed above). Such therapeutic endeavours will be enabled by better understanding of how escapees are expressed from the Xi.

As an important contributor to sex differences, the generation of an accurate list of escapees across tissues will be beneficial. However, such lists are not static, differing between individuals and tissues, and dependent on both the study approach and the thresholds chosen. Even the distinction between ”constitutive” and ”facultative” blurs with more data, suggesting multiple interacting contributors allowing for the expression from the Xi. We envision XCI as a continuum, with some genes always found to escape and others presenting rarely or in a tissue-specific manner. Further, the level of expression from the Xi varies across genes, and for the same gene, across tissues. In general, PAR1 genes show the highest Xi/Xa expression, but controlling mechanisms remain to be defined. With methodological advances, such as long-read sequencing that can support phasing and also provide a direct measure of skewing [148] as well as deep single-cell RNA-seq, our lists of escapees will continue to be expanded and refined. In the meantime, users need to be aware that there are multiple sources of lists, and that the contents of these lists are impacted by the thresholds used to call them. With such caveats considered, the inclusion of genes escaping XCI is an important consideration for the origin of sex differences.

## Appendix A

**Table A1:** Studies seeking elements differentiating between subject and escape genes. Studies are performed in human model systems unless otherwise noted.

Genomic Element	Source of XCI Status	Source of Element	Window Size	Key Results
**Repetitive elements: LINEs (L1s) enriched on X and in regions of genes subject to inactivation;** **SINEs (Alus) less prevalent on X but enriched at genes escaping from XCI**	XCI status from [149]	RepeatMasker for X to autosomes	Genomic segments ~200 kb	X was enriched 2-fold for L1 repetitive elements; escapee-containing regions were significantly reduced in L1 content compared with subject-containing regions [150]
	XCI status from [3,149]	RepeatMasker for X across evolutionary strata	TSS ± 50, 100 kb ^1^	LINEs were significantly enriched around subject genes, while Alu elements and short motifs containing ACG/CGT were significantly enriched around escapees ^2^ [151]
	XCI status from [3]	Oligomer enrichment analysis focused on Xp22	TSS ± 50, 100, 250 kb	38% of oligomers overrepresented in escapee-containing regions were located in Alu repeats, while 64% of the oligomers overrepresented in subject-containing regions were within L1 repeats ^2^ [152]
	DNAm predicted XCI status of autosomal genes in X; auto some translocations	DNA sequence features and epigenetic markers from ENCODE	TSS ±5, 15 kb, transcribed genic region	Alu elements were enriched at autosomal genes that escaped from inactivation while L1s were enriched at subject genes ^2^ [132]
	DNAm predicted XCI status of autosomal genes in X; autosome translocations	Motif enrichment plus RepeatMasker for inactivated vs active autosome genes	Gene ±50 kb	Motifs mapping to primate-specific L1s were enriched around both autosomal and X-linked genes silenced by XCI; *Alu* elements were enriched around autosomal genes that escape XCI ^2^ [133]
	RNA-seq detected “not-silenced” mouse genes with inducible *Xist*	RepeatMasker for mouse chromosomes X, 8, and 12	TSS ± 500 kb	Escape genes clustered in LINE-poor regions of chromosomes X, 12, and 8; SINE enrichment correlated with escape genes on both chromosomes X and 12 [134]
**Boundary elements: play a role at transition regions involved in maintaining both inactive and escape domains (CTCF, TAD-like structures)**	RNA-seq in mouse Patski cells and F1 hybrid brain with skewed XCI	ChIP-seq in mouse Patski cells and F1 hybrid brain with skewed XCI	Sliding 500 kb, step size 1 kb	CTCF binding was reduced on the Xi vs Xa except at escape regions, and was denser in brain compared to the Patski cell line, possibly contributing to a more compartmentalized structure of the Xi and fewer escape genes in brain compared to the cell line [144]
	RNA-seq in mouse Patski cells and F1 hybrid brain with skewed XCI	TF motifs and Chip-seq peaks in mouse Patski cells and F1 hybrid brain with skewed XCI	Escape domains	Escape gene domains are flanked by convergent arrays of CTCF binding sites present on both the Xa and Xi, often with cohesin subunit RAD21; facultative escapees showed differences in Xi CTCF binding dependent on their XCI status in various cells/tissues [139]
	RNA-seq in mouse trophoblast stem cells (TSCs, imprinted XCI)	ChIP-seq in TSCs	Gene ± 5 kb	Moderate positive correlation between CTCF binding and Xi expression; the majority of Xi CTCF peaks were located at sites shared with the Xa [153]
**Escape elements:** **Local control of escape from XCI (CTCF and other transcription-related factors)**	RNA-seq detected “not-silenced” mouse genes with inducible *Xist*	ChIP-seq of male mouse ESC X to autosomes	TSS ± 4 kb	Enrichment of CTCF at the TSSs of “not silenced” X-linked genes relative to partially affected and fully silenced genes; enrichment was notably absent for autosomal genes escaping XCI [134]
	RNA-seq of mouse ESC to NPC differentiation	ChIP-seq and ATAC-seq in mouse ESCs	≤5 kb from TSS, and >5 kb from TSS	51% of Xi accessible sites were <5 kb from a promoter compared to ~35% on the Xa, suggesting that escape is often regulated through promoter-proximal sites; most ATAC–seq peaks on the Xi were found at CTCF sites [112]
	PRO-seq in inducible *Xist* female mouse ESC line	Random forest model to predict XCI status based on biological peaks and genome features in male ESCs	Promoters or gene body regions	Top 10 features important for classifying escape included distance to *Xist* locus, gene density, RING1B, TAF1, MLL2, HDAC2, ESRRB, HDAC1, TET1, and E2F1; CTCF was not a discriminating feature at promoter, but signal was significantly enriched at enhancers of escape genes [154]
	Escape TSSs called using FANTOM5 CAGE data in multiple cell types; PAR excluded	TF motif and ChIP-seq peaks from primary cells, tissues and transformed cell lines	TSS ± 500 bp	Over-representation of YY1 around escape TSSs (X-linked and autosomal from X;autosome translocations); additional over-represented TFs at escape include PAX5, MYC, TBLR1, FOXM1, MAZ, ATF2, CTCF, CHD1 and SIN3A when compared to subject genes [155]
	55 constitutive escape genes [40]; variable escape and PAR were excluded	ReMAP ChIP-seq peaks in female GM12878	TSS ± 500 bp ^1^	Five TFs (ZFP36, NIPBL, MYB, STAT1, and HSF1) were enriched around escape compared to subject genes, and in general the number of TFs binding per gene was depleted for subject genes compared to escape and autosomal genes [142]

^1^ study considered other window sizes, most significant is listed. ^2^ study considered other repeat elements; most significant differences in subject and escape are described.
